# A Novel Fractional Order Model for the Dynamic Hysteresis of Piezoelectrically Actuated Fast Tool Servo

**DOI:** 10.3390/ma5122465

**Published:** 2012-11-23

**Authors:** Zhiwei Zhu, Xiaoqin Zhou

**Affiliations:** School of Mechanical Science and Engineering, Jilin University, Changchun 130022, China; E-Mail: wsjdzzw-jx@163.com

**Keywords:** fast tool servo, piezoelectric actuation, dynamic hysteresis nonlinearity, fractional order calculus, linearized hysteresis model

## Abstract

The main contribution of this paper is the development of a linearized model for describing the dynamic hysteresis behaviors of piezoelectrically actuated fast tool servo (FTS). A linearized hysteresis force model is proposed and mathematically described by a fractional order differential equation. Combining the dynamic modeling of the FTS mechanism, a linearized fractional order dynamic hysteresis (LFDH) model for the piezoelectrically actuated FTS is established. The unique features of the LFDH model could be summarized as follows: (a) It could well describe the rate-dependent hysteresis due to its intrinsic characteristics of frequency-dependent nonlinear phase shifts and amplitude modulations; (b) The linearization scheme of the LFDH model would make it easier to implement the inverse dynamic control on piezoelectrically actuated micro-systems. To verify the effectiveness of the proposed model, a series of experiments are conducted. The toolpaths of the FTS for creating two typical micro-functional surfaces involving various harmonic components with different frequencies and amplitudes are scaled and employed as command signals for the piezoelectric actuator. The modeling errors in the steady state are less than ±2.5% within the full span range which is much smaller than certain state-of-the-art modeling methods, demonstrating the efficiency and superiority of the proposed model for modeling dynamic hysteresis effects. Moreover, it indicates that the piezoelectrically actuated micro systems would be more suitably described as a fractional order dynamic system.

## 1. Introduction

Piezoelectric actuators (PEA), which possess superior advantages of high frequency response, nanometer displacement resolution, high stiffness and miniature size, have been extensively employed in micro/nano electromechanical systems, especially in fast tool servo (FTS) systems for micro/nano fabrications of freeform surfaces and functional structured surfaces [1,2,3,4]. However, due to the intrinsic friction within the material crystals of the PEA, there are always intrinsic hysteresis nonlinearities when voltages are employed as the excitations. The hysteresis effects of piezoelectric materials would significantly deteriorate the positioning performances of the cutting tool of the FTS and even lead to the instability of such servo systems, consequently limiting developments of FTS based micro/nano machining [5,6].

To enhance positioning accuracy and compensate hysteresis nonlinearities of this sort of FTS, closed-loop control approaches with various control strategies have been extensively proposed [2,4,7,8]. Generally, in these controller design procedures, the behaviors of FTS were described by simplified linear second order dynamics models, ignoring hysteresis nonlinearities. However, these control strategies should not achieve excellent positioning performances attributing to unmodeled nonlinearities. Moreover, these simplified models should strongly block the developments of model based control or compensation strategies. Therefore, accurately modeling the dynamic behaviors of the FTS with respect to the hysteresis nonlinearities should be essential for both optimum determination of controller parameters and developments of model based compensation strategies. Moreover, due to the reason that the trajectory of FTS should contain a large number of harmonic components with different amplitudes and frequencies [9,10], the complicated rate-dependent behaviors of hysteretic effects should be precisely modeled. As for the hysteresis effects of piezoelectrically actuated FTS, for sake of completeness, Wang *et al.* (2008) simply applied the Preisach model to FTS [11]; Ting *et al.* (2011) designed a piezoelectrically actuated cutting system which was similar to FTS, a typical dynamic Preisach model was employed to form the feedforward compensator [12]. As criticized in [13], the process failed to describe the dynamics aspects of the PEA, hence it would not be accurate enough to enhance the positioning performances.

Although little efforts have been devoted to the modeling and compensating for hysteresis effects of FTS, both feedback control and inverse model based feedforward compensation (IMFC) approaches have been proposed for hysteresis effects of piezoelectrically actuated mechanisms, such as nano-positioning stages, probe tips of atomic force microscope, and so on [12,14,15,16,17,18,19]. Generally, all those approaches should strongly depend on accurate models of the plants. Motivated by this, extensive mathematical models for dynamic hysteresis of PEA have been developed. However, almost all of these models are established based on complex nonlinear operators. As for the aspect of controller design processes, these nonlinear operators would significantly limit the implementations of well-developed analysis and control theories of linear systems. As for another aspect of the IMFC, these complex nonlinear operators will add difficulties to the calculation of model inversions and even lead to ill solutions [12,18,20]. In facing this dilemma, it is essential to develop a linearized model for accurately describing behaviors of piezoelectrically actuated FTS systems.

In this paper, the Fractional Order Calculus (FOC) theory is introduced to develop a dynamic hysteresis model to describe the rate-dependent hysteresis nonlinearities of PEAs. ] Then, a linearized fractional order dynamic hysteresis (LFDH) model for piezoelectrically actuated FTS systems is proposed and discussed. A brief review of related work is presented in Section 2. In Section 3, the basic definitions and properties of FOC theory are introduced. The modeling process and the properties of the proposed HFM are further given in Section 4. Section 5 focuses on the conduction of experiments to verify the effectiveness of the proposed model. The main conclusions and the future work of this paper are drawn in Section 6.

## 2. A Brief Review of Related Work

Mrad and Hu and Hu *et al.* extended the classical Preisach model to describe the rate-dependent behaviors of hysteresis by use of an explicit weighting function with respect to the average change rate of the input signal [21,22,23]. To capacitate the Preisach model to represent the dynamic behaviors of controlled PEA, Yu *et al.* modified the weighting function to be dependent on the variation rates of input signal; To avoid the ill-behaviors caused by the great variations of input signal, an adjustment function with respect to the variation rate of input signal was introduced, which should be fitted through experimental data [24]. Recently, various rate-dependent Prandtl–Ishlinskii (PI) elementary operators have been introduced to model dynamic hysteresis effects. Ang *et al.* proposed a modified dynamic PI model, the rate-dependent hysteresis was modeled by the rate-dependent weighting values which were derived from the linear slope model of the hysteresis curve [25,26]. Janaideh *et al.* introduced a dynamic threshold, which was a function of input variation rates, the relationship between the threshold and the variation rate of input signal is in the logarithmic form to describe the essential characteristics of the hysteresis [27,28,29]. In both the generalized Preisach model and the PI model, the hysteresis loops were modeled by the sum of a number of elementary operators, and the rate-dependent behaviors were further described by modified dynamic weighting values, which were often functions of the derivation of input signal. The main disadvantage of these modeling methods is that they have a large number of parameters to be identified, which may limit their applications in real-time control.

Besides the well-known Preisach model and PI model, neural network (NN) based methods have also been extensively employed to model the dynamic hysteresis effects. Dong *et al.* employed a feedforward NN to model the hysteresis of the PEA, the variation rate was used to construct the expanded input space [30]. Zhang and Tan proposed a parallel hybrid model for the rate-dependent hysteresis, a neural submodel was established to simulate the static hysteresis loop, meanwhile, first-order differential operators with time delays based submodel were employed to describe the dynamics of the hysteresis [31]. However, there exist inherent defects of NN based modeling, which can be summarized as follows: (a) There is no universal rules to optimally determine the structure of the NN; (b) NN has the shortcomings of overfitting and sinking into local optima [32]; (c) The capacities of fitting and prediction could not be well balanced.

Some other novel mathematical models for dynamic hysteresis have been proposed. For instance, by transforming the multi-valued mapping of hysteresis into a one-to-one mapping, Deng and Tan proposed a nonlinear auto-regressive moving average with exogenous inputs (NARMAX) model to describe the dynamic hysteresis [33]. Similarly, Wong *et al.* formulated the modeling as a regression process and proposed the online updating least square support vector machines (LS-SVM) model and the relevance vector machine (RVM) model to capture the dynamic hysteretic behaviors [32]. Nevertheless, a compromise should be made between the modeling accuracy and the updating time, which meant that it would be challenged to apply it for high-frequency working conditions. Rakotondrabe *et al.* modeled the dynamic hysteresis to be a combination of the static Bouc-Wen model and a second-order linear dynamic part [34]. In [35] and [36], Gu and Zhu proposed an ellipse-based hysteresis model where the frequency and amplitude of the input signal was modeled by adjusting the major and minor axes and orientation of the ellipse. However, the model parameters were difficult to be determined to well describe and predict the dynamic hysteresis characteristics, and the ability of describing responses to the input signals with multi-frequencies would be limited.

Fractional order calculus (FOC) theory, which is a generalization of the conventional calculus theory, has found a rapidly increasing application in various fields [37,38,39]. It has been widely believed that FOC can be used to describe a real process more accurately and more flexibly than classical methods [38,39,40]. A typical implementation of FOC is the description of dynamic properties of visco-elastic materials [41,42]. Motivated by the fractional order models for visco-elastic materials, Sunny *et al.* proposed two models to describe the resistance-strain hysteresis of a conductive polymer sample by combining a series of fractional/integer order functions [43]. However, both the developed models contained too many parameters to be identified and the existing hysteresis phenomenon was different from that of PEAs. Guyomar *et al.* described the ferroelectric hysteresis dynamics based on fractional order derivatives covering a wide range of frequency bandwidth [44,45]. In this method, the fractional order derivative term was employed to represent the viscous-like energy loss, and the derivative order was especially set as 0.5. Although the fixed order would present the unique characteristics of fractional calculus, it would significantly decrease the flexibility of the model and block the application of this method. Similar with the work presented by Sunny *et al.*, the hysteresis between the electrical polarization and the mechanical strain was also much different from that of the PEA. However, all these results have demonstrated the potentials of fractional order models in modeling both the static and the dynamic hysteresis behaviors, and provided a fresh idea towards this topic.

## 3. A Preliminary to Fractional Order Calculus

Fractional calculus is a generalization of conventional integration and differentiation to the non-integer order with the fundamental operator ${\;}_{t_{0}}D_{t}^{a}f(t)$ which is defined as:

$${\;}_{t_{0}}D_{t}^{\alpha }f(t)=\{\begin{array}{ll} d^{\alpha }/dt^{\alpha }, & \text{Re}(\alpha )>0\\ 1, & \text{Re}(\alpha )=0\\ \int_{t_{0}}^{t}{\left(d\tau \right)}^{\alpha }, & \text{Re}(\alpha )<0 \end{array}$$

where *t_0_* and *t* are the lower and upper limits of the operation, respectively; *α* is the order, α∊R, but *α* could also be a complex number.

There exist several well-known definitions of FOC operations, such as Grunwald—Letnikov (G-L) definition and Riemann—Liouville (R-L) definition [40,42,46]. The R-L definition would be more suitable for analytical discussions and is given as:

(1) $${\;}_{t_{0}}D_{t}^{\alpha }f(t)=\frac{1}{\Gamma (n-\alpha )}\frac{d^{n}}{dt^{n}}\int_{t_{0}}^{t}\frac{f(\tau )}{{\left(t-\tau \right)}^{\alpha -n+1}}d\tau ,n-1<\alpha <n$$

In this paper, the G–L definition is employed to directly carry out numerical computation of fractional order operators, which can be given as:

(2) $${\;}_{t_{0}}D_{t}^{\alpha }f(t)=\underset{h\to 0}{\text{lim}}h^{-\alpha }\sum_{j=0}^{\left[(t-t_{0}/h)\right]}{\left(-1\right)}^{j}\frac{\Gamma (\alpha +1)}{\Gamma (\alpha -j+1)\Gamma (j+1)}f(t-jh)$$

where Γ( ) is the Gamma function, *h* is the calculation step, *n* is an integer.

As shown in Equation (1) and Equation (2), if *f(t)* is an analytical function of *t*, its fractional derivative ${\;}_{0}D_{t}^{a}f(t)$ would be an analytical function of *t* and *α*, this may increase the flexibility of FOC for representing the modeled objects. Meanwhile, it is more evident from the G-L definition that the weight of *f*(*t − jh*) is decreasing with the increase of *j*. It indicates that the fractional order operation possesses variable memories with respect to different temporal intervals. Thus, to take advantage of the memory effects of FOC operations, the nonlocal memory-dominant nature of hysteresis nonlinearity could be well described and the hysteresis system would be more suitable to be described as a fractional order dynamic system.

On the other hand, both fractional differentiation and integration are linear operations, which satisfy:

(3) $${\;}_{t_{0}}D_{t}^{\alpha }[af(t)+bg(t)]=a\cdot {\;}_{t_{0}}D_{t}^{\alpha }f(t)+b\cdot {\;}_{t_{0}}D_{t}^{\alpha }g(t)$$

where *a* and *b* are constants, *g*(*t*) is another analytical function of *t*.

For zero initial conditions, the Laplace transform of both G-L and R-L definitions can be written as [40,47]:

(4) $$L[{\;}_{0}D_{t}^{\alpha }f(t)]=s^{\alpha }L[f(t)]=s^{\alpha }F(s)$$

A typical fractional order dynamic system can be described by a fractional differential equation of the following form [47]:

(5) $$a_{N}D^{\alpha_{N}}x(t)+a_{N-1}D^{\alpha_{N-1}}x(t)+\text{L}\;a_{0}D^{\alpha_{0}}x(t)\;=b_{M}D^{\beta_{M}}u(t)+b_{M-1}D^{\beta_{M-1}}u(t)+\text{L}\;b_{0}D^{\beta_{0}}x(t)$$

Taking Laplace transform of the two sides in Equation (5), the fractional-order linear time-invariant (LTI) system can also be represented by the following transfer function:

(6) $$G(s)=\frac{X(s)}{U(s)}=\frac{\sum_{m=0}^{M}b_{m}s^{\beta_{m}}}{\sum_{n=0}^{N}a_{n}s^{\alpha_{n}}}$$

where $D^{\gamma }={\;}_{0}D_{t_{0}}^{\gamma }$, *a_n_* and *b_m_* are constants, *α_n_* and *β_m_* are arbitrary real numbers.

In the fractional order linear and time-invariant (LTI) system as shown in Equation (6), the Matignon’s stability theorem is usually employed as the stability criterion, which says [40,48]:

The fractional transfer function *G*(*s*) = *X*(*s*)/*U*(*s*) is stable if and only if the following condition is satisfied in *σ*-plane:

(7) $$\left|\text{arg}(\sigma )\right|>q\frac{\pi }{2},\forall \sigma ∊C,U(\sigma )=0$$

where 0 < *q* < 1, denotes the fractional commensurate order; *σ*: = *s^q^*. When *σ* = 0 is a single root of *U*(*s*), the system could not be stable.

## 4. Fractional Order Hysteresis Model of the FTS

### 4.1. Dynamic Model of the FTS Mechanism

**Figure 1:**
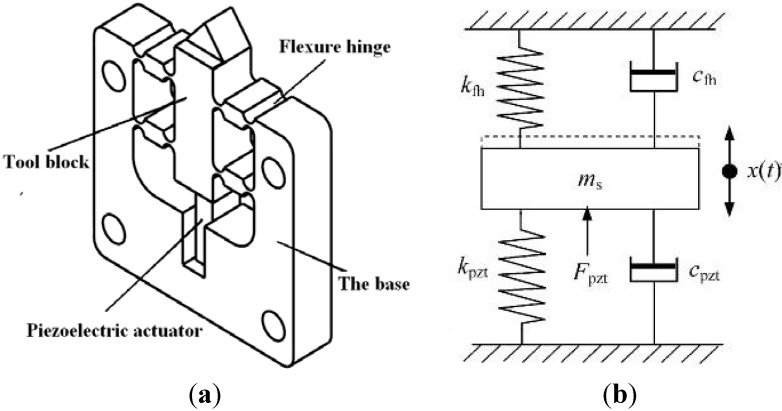
The schematic diagram of FTS structure and its equivalent dynamic model: (**a**) The schematic diagram of FTS structure; (**b**) The equivalent dynamics model.

Figure 1a illustrates the mechanical structure of a typical piezoelectrically actuated FTS, the flexure based mechanical body of the FTS is actuated by a PEA and supported by a group of parallel and symmetric flexure hinges. From the dynamics point of view, the FTS mechanism can be equivalent as a damped mass-spring system as shown in Figure 1b [49,50]. We assume that the PEA and the tool block did not separate during rapid expansions and retractions of the PEA considering the pre-loading effects, and the displacement of the PEA is equivalent to that of the mechanism.

Based on Newton’s second law of motion, the differential equation of motions for the FTS can be expressed as [49,51]:

(8) $$m_{\text{s}}\ddot{x}(t)+(c_{\text{pzt}}+c_{\text{fh}})\dot{x}(t)+(k_{\text{pzt}}+k_{\text{fh}})x(t)=F_{\text{pzt}}(t)$$

(9) $$F_{\text{pzt}}(t)=nd_{33}k_{\text{pzt}}u_{\text{pzt}}(t)$$

where *k*_fh_ and *k*_pzt_ are the equivalent stiffness of the flexure-based mechanism and the PEA, respectively; *c*_fh_ and *c*_pzt_ are the equivalent damping coefficients of the flexure-based mechanism and the PEA, respectively; *m*_s_ is the equivalent mass of the mechanism; *F*_pzt_(*t*) is the driving force of the PEA (see Figure 1); *n* is the number of layers in the PEA; *d*_33_ is the piezoelectric constant; *u*_pzt_(*t*) is the voltage applied to the PEA; *x*(*t*) denotes the output of the FTS mechanism.

**Figure 2:**
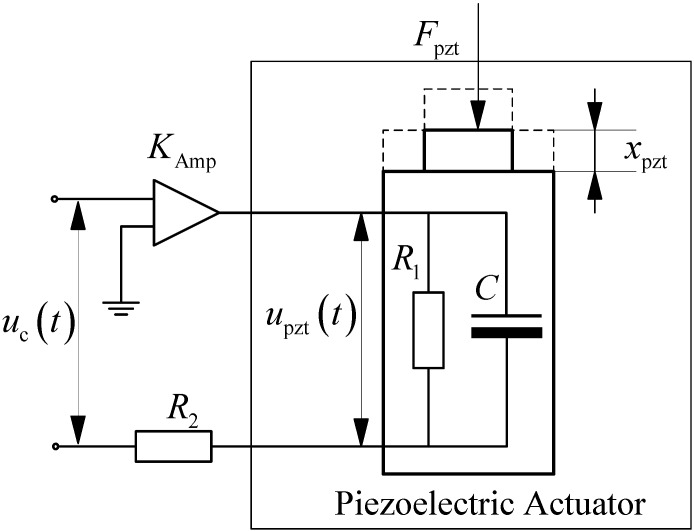
Equivalent driving circuit of the piezoelectric actuators (PEA).

From an electrical circuit point of view, the capacitance–resistance parallel connected equivalent circuit could be employed to represent the PEA under dynamic working conditions [52], and the electrically driven circuit can be illustrated in Figure 2. The relationship between the actual voltage *u*_pzt_(*t*) applied to the PEA and the control signal *u*_c_(*t*) can be expressed as follows:

(10) $$R_{2}[C\frac{du_{\text{pzt}}(t)}{dt}+\frac{u_{\text{pzt}}(t)}{R_{1}}]+u_{\text{pzt}}(t)=K_{\text{Amp}}nd_{\text{33}}u_{\text{c}}(t)$$

where *R*_1_ and *C* are the equivalent resistance and capacitance of the PEA, *R*_2_ is the equivalent resistance of the amplifier, *K*_Amp_ is the nominal amplification factor of the power amplifier.

### 4.2. Hysteresis Model of the FTS

Generally, actuating forces generated by PEA would well follow the governing law in Equation (9) when hysteresis effects are ignored. To describe the hysteresis nonlinearity between actual voltages applied to the PEA and the corresponding displacements of the PEA, a hysteresis force model (HFM) is proposed and mathematically described by a linear fractional order differential equation. From the view of excitations applied to the PEA, the HFM can be given as:

(11) $$\left\{ \begin{array}{l} F_{\text{H}}(t)=nd_{33}k_{\text{pzt}}[u_{\text{pzt}}(t)+\kappa \cdot D^{\eta }u_{\text{pzt}}(t)]\\ 0<\eta <1,\kappa >0,\kappa ∊\text{ℜ} \end{array}\right.$$

From Equation (9), Equation (11) can be further rewritten as:

(12) $$\left\{ \begin{array}{l} F_{\text{H}}(t)=F_{\text{pzt}}(t)+\chi \cdot D^{\lambda }F_{\text{pzt}}(t)\\ 0<\lambda <1,\chi >0,\chi ∊\text{ℜ} \end{array}\right.$$

where *F*_H_(*t*) denotes the hysteresis force, *κ* and *χ* are constant gains, *η* and *λ* are differential orders of the excitation voltage and the driving force, respectively.

From Equation (11) and (12), the relationship between the excitation voltage and the actual driving force yields:

(13) $$F_{\text{pzt}}(t)+\chi \cdot D^{\lambda }F_{\text{pzt}}(t)=nd_{33}k_{\text{pzt}}[u_{\text{pzt}}(t)+\kappa \cdot D^{\eta }u_{\text{pzt}}(t)]$$

Taking Laplace transform of two sides in Equation (13) yields:

(14) $$H(s)=\frac{F_{\text{pzt}}(s)}{U_{\text{pzt}}(s)}=\frac{nd_{33}k_{\text{pzt}}[1+\kappa \cdot s^{\eta }]}{1+\chi \cdot s^{\lambda }}$$

where *F*_pzt_(*s*) and *U*_pzt_(*s*) denote the Laplace transform of *F*_pzt_(*t*) and *u*_pzt_(*t*), respectively; *s* denotes the Laplace operator.

Taking Laplace transform of two sides in Equation (8) and Equation (10), and combining Equation (14) yields the transfer function of the piezoelectrically actuated FTS involving its dynamic hysteresis effects:

(15) $$P(s)=\frac{X(s)}{U_{\text{c}}(s)}=\frac{1}{\tau s+1}\frac{K_{\text{M}}}{s^{2}+2\text{ξ}\omega_{n}s+\omega_{n}^{2}}\frac{K_{\text{P}}+\Theta \cdot s^{\eta }}{1+\chi \cdot s^{\lambda }}$$

where *τ*= *R*_1_*R*_2_*C*/(*R*_1_ + *R*_2_), 2ξ*w_n_* = (*c*_pzt_ + *c*_fh_)/*m*_s_, $\omega_{n}^{2}$ = (*k*_pzt_+ *k*_fh_)/*m*_s_, *K*_M_ = *K*_Amp_*nd*_33_*R*_1_/[*m*_s_(*R*_1_ + *R*_2_)], *K*_P_ = *nd*_33_*k*_pzt_ and Θ = *κnd*_33_*k*_pzt_.

### 4.3. Properties of the Hysteresis Force Model

During the FTS assisted turning process, the FTS is used to translate the cutting tool in and out of the workpiece several times per one revolution according to the geometrical characteristics of the desired workpiece surfaces [9,53,54,55]. Consequently, the tool trajectories can be decomposed into a sum of harmonics of the spindle rotation from the view of Fourier series expansion. Thus, the voltage applied to the PEA can be written as:

(16) $$u_{\text{pzt}}(t)=u_{0}+\sum_{k=1}^{J}{\dot{\text{ζ}}}_{k}e^{j(k\omega_{0}t+\varphi_{k})}$$

Where $j=\sqrt{-1}$, *u*_0_ is the constant item; ω_0_ is the frequency of the spindle rotation; *ϕ*_k_ and *ζ*_k_ are the phase shift and Fourier expansion coefficient, respectively.

Following the HFM given in Equation (11), the response of the HFM would be:

(17) $$F_{\text{H}}(t)=nd_{33}k_{\text{pzt}}[u_{\text{pzt}}(t)+\frac{u_{0}t^{-\eta }}{\Gamma (\eta )}+\kappa \sum_{k=1}^{J}{\dot{\text{ζ}}}_{k}{\left(k\omega_{0}\right)}^{\eta }e^{\sum_{k=1}^{J}j(k\omega_{0}t+\varphi_{u}+\frac{\pi \eta }{2})}]$$

**Figure 3:**
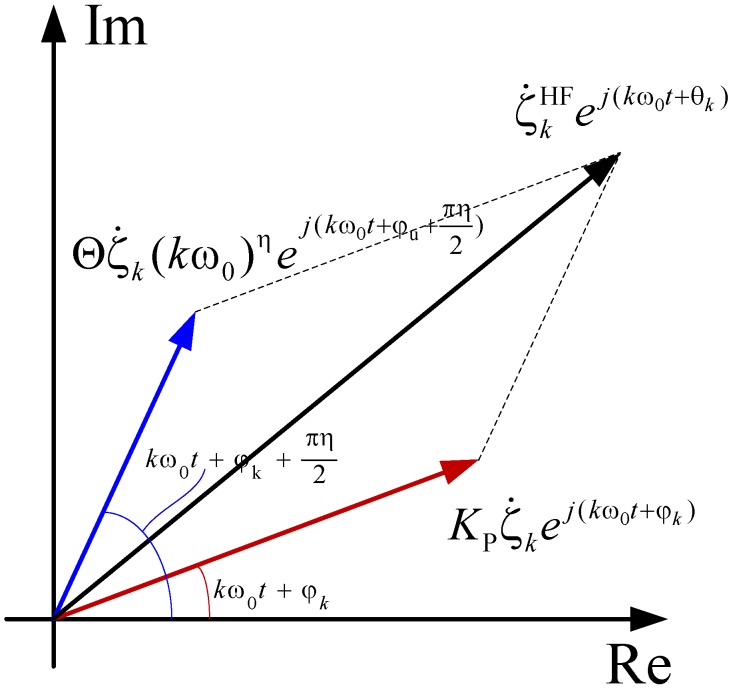
Calculation process in the complex plane.

As seen in Equation (17), the hysteresis force comprises three items, namely the original signal, the attenuation term and the modulation item. As for a given harmonic component with a specified frequency, the superposition process can be illustrated in Figure 3, where $\zeta_{k}^{\text{HF}}$ and *θ_k_* denote the amplitude and phase shift after superposition.

It should be noticed that Cruz-Hernández and Hayward proposed a hysteresis control method based on phase shift method, they regarded the static hysteresis as the nonlinear phase lag which varied with the magnitude of a specified period signal [56]. Meanwhile, as discussed in Section 2, the dynamic hysteresis effects were often emphasized by variation rate dependent weighting values, However, from the diagrammatized results shown in Figure 3, the response of the HFM can be characterized by a nonlinear phase-shift and the nonlinear amplitude modulation, both depending on the frequency of the harmonic function, the gains and the differential order. Attributing to the unique frequency-dependent effects, the dynamic hysteresis behaviors of PEAs could be better described by the proposed fractional order dynamic model.

**Figure 4:**
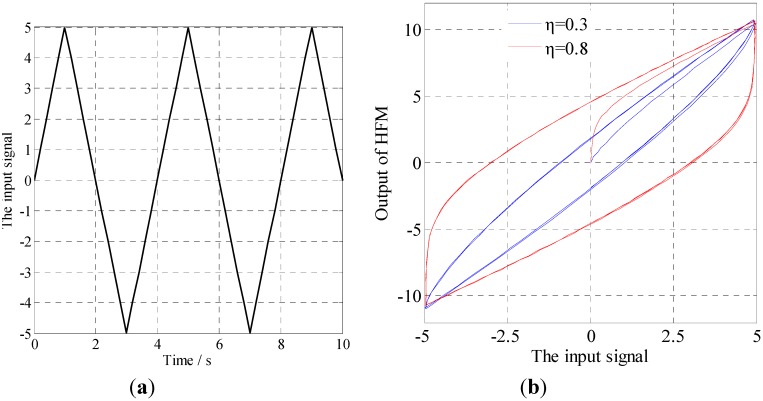
Characteristics of the HFM: (**a**) The input signal; (**b**) The relationship between the input and the response.

To give more visible results of the frequency dependent effects of the HFM, a typical triangle signal involving harmonic components with various frequencies is employed to generate the hysteresis force where *K*_p_ = Θ = 1. Figure 4a gives the employed triangle signal, and Figure 4b shows the relationship between the generated hysteresis force and the input signal. As is evident from the results shown in Figure 4, the nonlinear hysteresis loop is well generated. Furthermore, it can be deduced that various sorts of hysteresis loops would be well generated by choosing different parameters.

## 5. Experiment Results and Discussion

### 5.1. Experiment Setup

**Figure 5:**
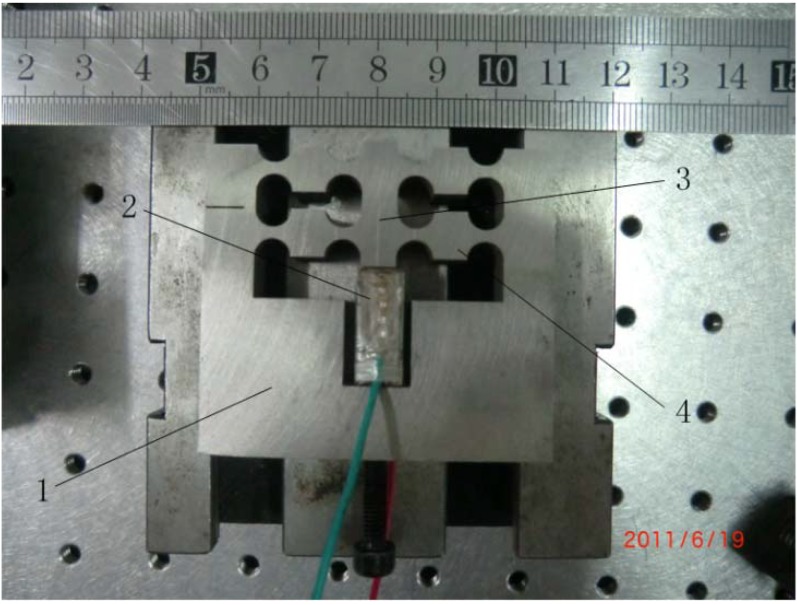
Photographic of the FTS mechanism.

Notes: 1. The base; 2. The piezoelectric actuator; 3. The tool holder; 4. The flexure hinges.

The authors of this paper carefully designed a short stroke FTS for ultra-precision diamond turning in Reference [57], the photographic of the designed FTS is presented in Figure 5. It consists of four main parts, namely: the base, the PEA, the tool holder and the flexure hinges. Under working conditions, the tool holder is actuated by the PEA and guided by a group of parallel and symmetric flexure hinges, which are designed as circular notch-type hinges. In the design process, the position and dimension parameters of the flexure hinges were determined by a multi-objective optimum approach to achieve comprehensive optimum performances.

**Figure 6:**
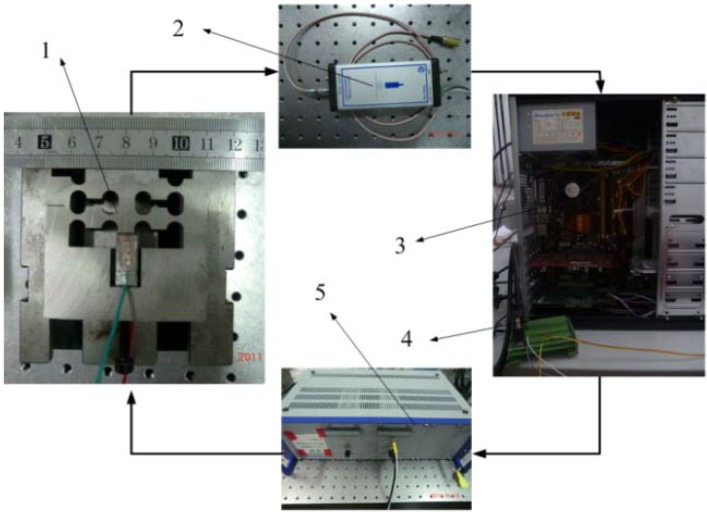
Schematic of the experimental system.

Notes: 1. The FTS mechanism; 2. The capacitive transducer; 3. The computer equipped with the data-acquisition card; 4. The data-acquisition card from ADLINK; 5. The power amplifier module PI E-617.

Figure 6 illustrates the test and measurement equipment of the experiment part. As shown in Figure 6, a Pentium computer equipped with a data-acquisition card is used to generate the control signal for the PEA. The generated signal is converted through the data-acquisition card from ADLINK sampled at 5 kHz and then amplified by an amplifier module PI E-617 with a nominal amplification factor 10 ± 0.1. The amplified signal is then implemented on a piezoelectric stack actuator (Polytec PI, Inc., Karlsruhe, Germany), which measures 7 mm × 7 mm × 18 mm. Capacity transducer based sensing methodology, which is frequently employed for trajectory tracking of FTS, is chosen for dynamic position measurements. The resolution of the high precision capacitive sensor is 0.0077% of the full stroke and its working bandwidth is up to 35 kHz, with an effective measurement range of 200 μm. The measured signal is converted to a digital signal by the data-acquisition card sampled at 10 kHz, and then gathered and stored in the computer for further analysis. To reduce external disturbances, all the experiments are carried out on a vibration-isolated air-bearing platform. The measurement noise of the testing system is 32 nm rms.

### 5.2. Parameter Estimation of the Model

Two typical micro-functional surfaces, namely sinusoidal array surface [10,58] and sinusoidal grid surface [1,53], are employed as the desired surfaces to validate the hysteresis model of the FTS. Considering the surface characteristics and the tool geometry, spiral toolpaths are determined, which will be detailed in our future work. As the main purpose of this paper is to verify the efficiency of the LFDH model, the generated toolpath signals are scaled and then directly utilized as control signals to actuate the FTS.

As shown in Equation (17), there exists an attenuation term in the hysteresis force if the excitation is not a pure harmonic function with zero-mean, which may lead to tendency errors in the model results. To compensate for tendency errors, a linear compensator is added which is defined as:

(18) Ψ(t)=ρt+δ

A least square based error function is used to estimate the model parameters, and defined as:

(19) $$E=\sum_{t_{m}=0}^{T}{\left[R_{\text{E}}(t_{m})-R_{\text{M}}(t_{m})\right]}^{2}$$

where *R*_E_(*t_m_*) is the measured displacement of the FTS from experiment at the time *t_m_*, and *R*_M_(*t_m_*) is the corresponding output of the proposed model.

Consequently, the parameter estimation procedure can be formalized as a constrained multi-dimensional optimization problem. To solve this problem, similar evolutionary optimization scheme as shown in Reference [59] is established to minimize the error function. The particle swarm optimization (PSO) based searching tool in [59] is replaced by an improved differential evolution (DE) algorithm, where a self-adaptive scheme of the control parameters of DE is employed to enhance the searching capacity of classical DE [60]. More details of the parameter estimation process would be given in our future work. The experimental data in the time interval [0,0.25] seconds in Case 2 is employed for the parameter estimation. Since the sampling rate for gathering data is set as 10 kHz, the calculation step in Equation (2) defaults to be 0.0001 s. The model parameters is then obtained and given in Table 1.

The stability of the LFHM highly depends on the differential orders, we employed the Matignon’s stability theorem to investigate the stability of the identified LFHM. As for the model, the denominator can be obtained as:

(20) $$D(s)=0.00289s^{3.811}+0.000418s^{3}+9.219s^{2.811}+1.337s^{2}+481282.557s^{1.811}+69779.6$$

**Figure 7:**
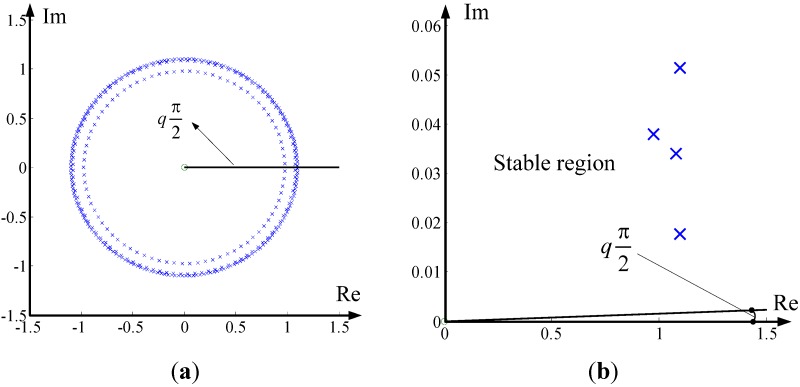
Pole positions with zoomed area: (**a**) pole positions; (**b**) zoomed plot.

The least common divisor of these differential orders is *q* = 0.001. The pole position plot is obtained as shown in Figure 7, and from the zoomed plot, it is immediately found that all the poles are located in the stable area, which means that the system is stable.

**Table 1:** Model parameters.

Parameters	Value
*K*_M_	1.71e + 8
*ζ*	0.031
*ω_n_*	1.28e + 4
*τ*	4.18 e − 4
*η*	0.756
*λ*	0.811
*K*_p_	0.192
Θ	5.923
*χ*	6.897
*ρ*	−0.140
*δ*	0.072

### 5.3. Validation of the Hysteresis Model

#### 5.3.1. Case 1

**Figure 8:**
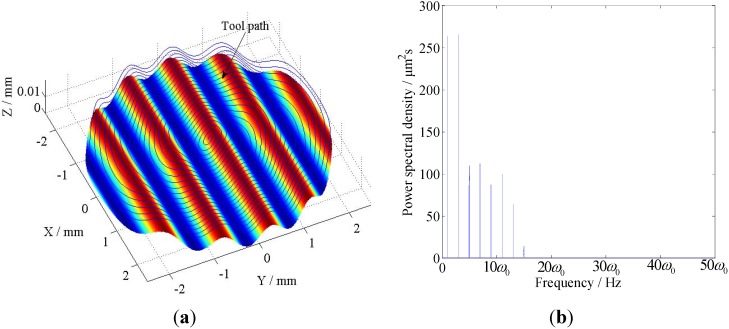
The sinusoidal surface and the PSD analysis of its tool path: (**a**) Schematic of the sinusoidal surface and its tool path; (**b**) PSD analysis of the tool path.

In this case, a sinusoidal profile of *z*(*x*,*y*) = 0.004*sin*(2*πx*) + 0.004 mm is employed as shown in Figure 8a. The power spectral density (PSD) of the toolpath after subtracting its mean value is illustrated in Figure 8b, where *w_0_* denotes the frequency of the spindle rotation. As shown in Figure 8b, the toolpath mainly consists of eight harmonic components with different frequencies.

**Figure 9:**
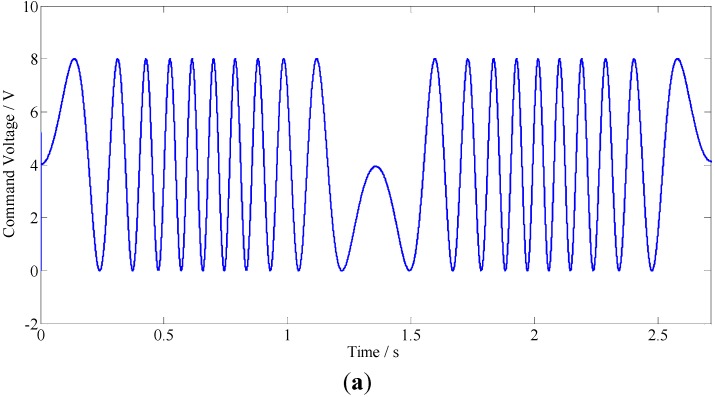

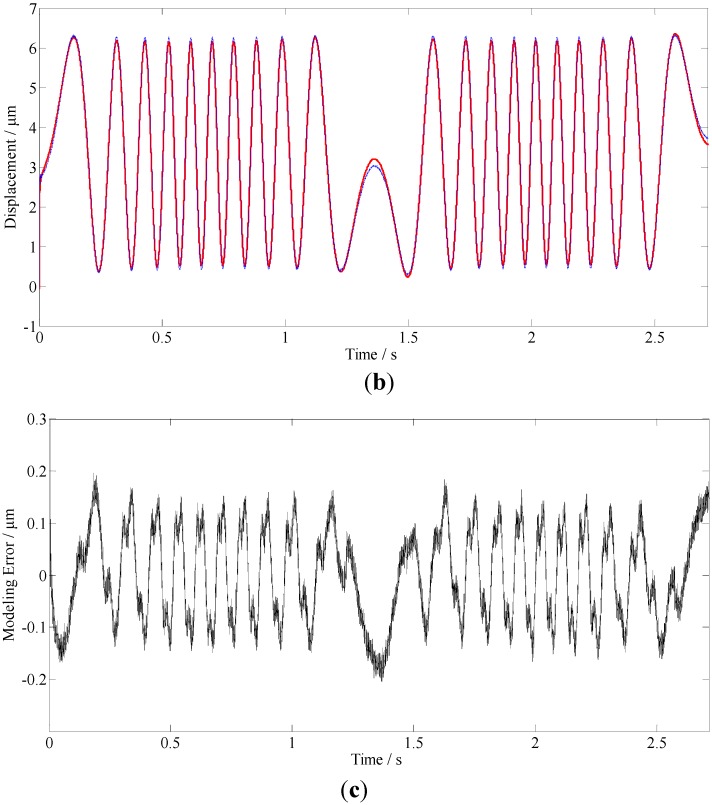
Modeling results (In Figure 9b, the red line and blue line denote the output of the model and the FTS, respectively.): (**a**) The control voltage; (**b**) Responses of the FTS and the proposed model; (**c**) The modeling error.

Figure 9a shows the command voltage applied to the PEA, the resultant responses of the FTS and the model are illustrated in Figure 9b, the modeling error is further given in Figure 9c. As shown in Figure 9b, there exists a constant motion shift of the FTS with zero input, it is about 0.35 μm. Fortunately, the motion shift is also well described by the proposed model. From the results shown in Figure 9c, the maximum modeling error is less than 0.2 μm, and the relative error in the steady state is less than ±2.5% of the full span range.

#### 5.3.2. Case 2

**Figure 10:**
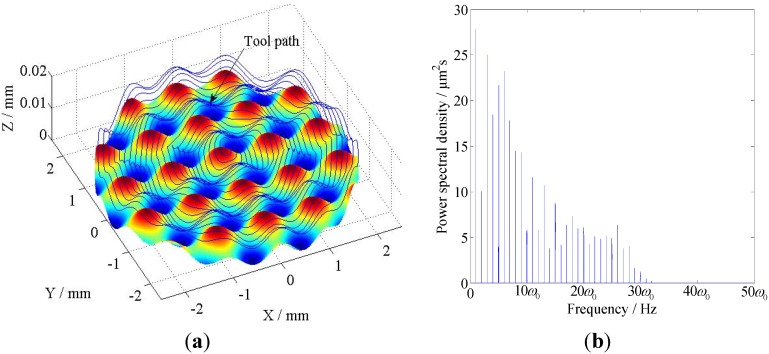
The sinusoidal grid surface and the PSD analysis of its tool path: (**a**) Schematic of the sinusoidal grid surface and its tool path; (**b**) PSD analysis of the tool path.

In this case, a sinusoidal grid surface of *z*(*x*,*y*) = 0.002*sin*(2*πx*) + 0.002*cos*(2*πx*) mm is employed. The surface is shown in Figure 10a, the PSD of the toolpath after subtracting its mean value is illustrated in Figure 10b. Comparing with the PSD results shown in Figure 8b, this toolpath consists of more harmonic components and covers a much wider range of frequencies.

**Figure 11:**
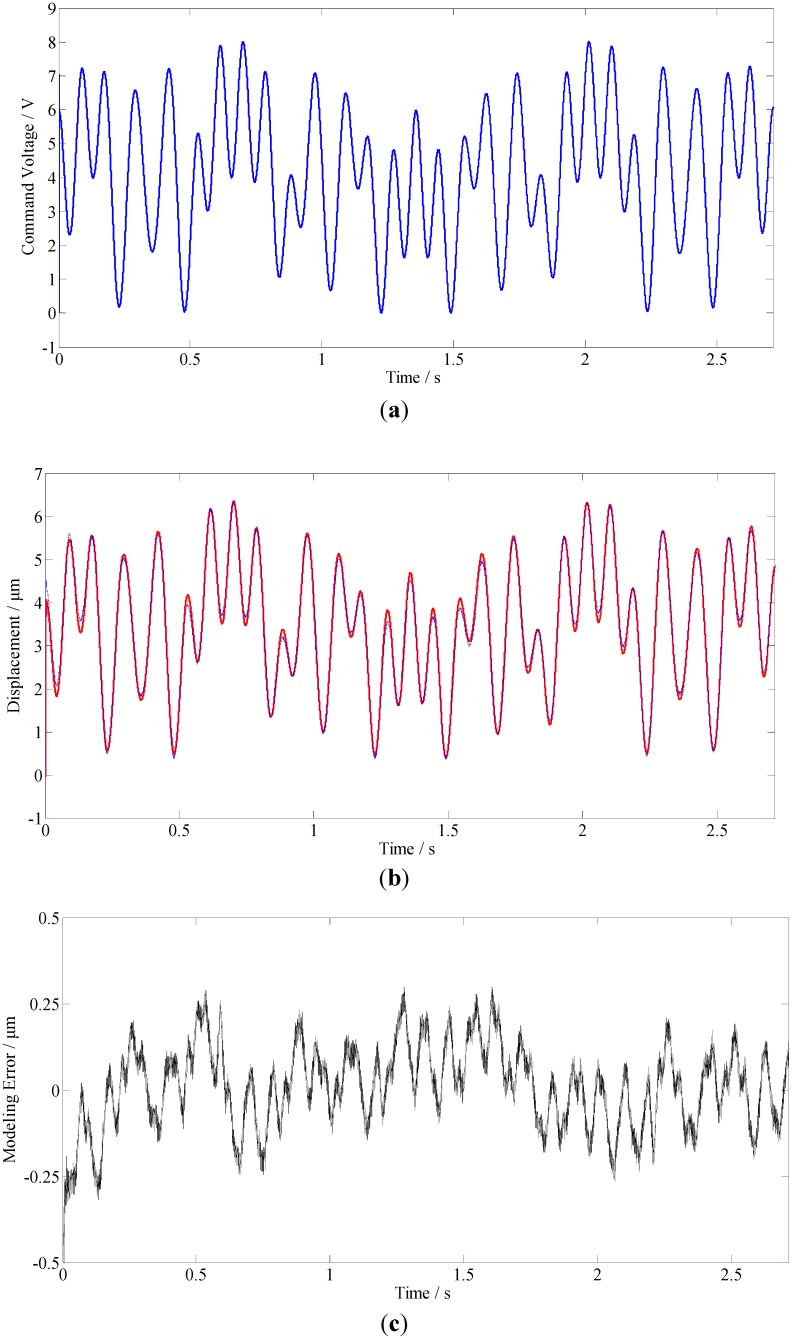
Modeling results (In Figure 11b, the red line and blue line denote the output of the model and the FTS, respectively.): (**a**) The control voltage; (**b**) Responses of the FTS and the proposed model; (**c**) Modeling errors.

Figure 11a shows the command voltage applied to the PEA, it is clear that it not only involves harmonic components with various frequencies but also involves amplitude variations in the control voltage. The resultant responses of the FTS and the model are illustrated in Figure 11b, the modeling error is further given in Figure 11c. As shown in Figure 11b, response generated by the proposed model agrees well with the actual response measured from the FTS mechanism. From the results shown in Figure 9c, the maximum modeling error is less than 0.25 μm, and the peak-to-peak value of the modeling error in the steady state is about 0.3 μm, which is less than ±2.5% of the full span range.

The results obtained in Cases 1 and 2 demonstrate that the proposed LFHM is efficient for modeling dynamic hysteresis nonlinearities, and the piezoelectric actuated FTS would be more suitable to be described as a fractional order dynamic system.

### 5.4. Comparison with Certain State-of-the-Art Modeling Methods

To verify the efficiency of the proposed LFDH model, the corresponding modeling errors were compared with errors of certain state-of-the-art modeling methods, including a generalized rate-dependent PI model (GPI) [29], a dynamic Preisach model (DPM) [23], the discrete ARMA-based dynamic hysteresis model (DARMA) [61], the conventional Bouc-Wen (BW) model and Non-symmetrical Bouc-Wen (NSBW) model [62], *etc*.. The comparison results are shown in Table 2. As for the GPI, the excitation is a harmonic signal with constant frequency and variable amplitude, while several separate experiments with harmonic excitations of different frequencies were conducted in [23], and the modeling error presented in Table 2 was at 800 Hz. As for the DARMA, the online estimation method based on trapezoid algorithm was employed to get a better identification of model parameters. The DARMA.a denotes the excitation signal with variable amplitude at 200 Hz, and the DARMA.b denotes a hybrid signal, which was a superposition of four sinusoidal signals with different frequencies, amplitudes and phase delays. As for the NSBW.a and BW.a, the excitation signal was a sinusoidal signal with a specified frequency and decreasing amplitude, while NSBW.b and BW.b denote a sort of non-periodic excitation signals. From the comparison results shown in Table 2, it is evident that the LFDH could describe the dynamic hysteresis behaviors more accurately, and it is of more excellent performance than these modeling methods.

**Table 2:** Comparison results of modeling error.

Model	Error
LFDH	±2.5%
GPI	±5.7%
DPM	6.4%
DARMA.a	±2.5%
DARMA.b	±5.6%
NSBW.a	±2.54%
NSBW.b	±2.76%
BW.a	±3.58%
BW.b	±3.96%

## 6. Conclusion

In this paper, a linearized mathematical model is proposed to describe dynamic hysteresis nonlinearities of a piezoelectrically actuated FTS. It features the following.

- Fractional order calculus (FOC) theory is introduced to establish a model for dynamic hysteresis nonlinearities, a fictitious hysteresis force is introduced and mathematically described by a fractional order differential equation. The hysteresis force model (HFM) can be characterized by nonlinear phase-shifts and nonlinear modulations of amplitudes, both mainly depending on input frequencies and differential orders. By choosing proper model parameters, the dynamic hysteresis effects could be well described.
- Combining the linear dynamics model of the FTS mechanism and the HFM, a linearized fractional order dynamic hysteresis (LFDH) model is proposed for the piezoelectrically actuated FTS system. The linearization feature of the LFDH model could make easier to implement the inverse dynamic control, and give an excellent playground for the well-developed linear control theories. Besides, certain accurate model assisted state-of-the-art control or compensation strategies for nonlinear systems would also be potential for implementing on the FTS systems.
- To verify the efficiency of the LFDH model, the toolpath signals for creating two typical micro-functional surfaces, which cover a wide range of frequencies, are scaled and utilized as command signals for a piezoelectrically actuated FTS. By means of an evolutional scheme, the parameters of the model are estimated. The modeling errors in the steady state are all less than ±2.5% of the full span range, which is much smaller than the modeling errors of certain state-of-the-art modeling methods. The results demonstrate that the proposed linear model is of more excellent performance for modeling dynamic hysteresis nonlinearities, and the piezoelectrically actuated micro-systems would be more suitable to be described as a fractional order dynamic system.

The following works need to be carried out in the future to further develop the proposed LFDH model:

- A more efficient parameter estimation method should be constructed to determine the best parameters of the LFDH model.
- The inverse model based hysteresis compensation approach should be further implemented with FTS to enhance the positioning accuracy of the cutting tool.
